# P023 Posterior reversible encephalopathy syndrome in historical triple-positive anti-phospholipid syndrome: diagnostic complexity and individualised anticoagulation

**DOI:** 10.1093/rap/rkag113.053

**Published:** 2026-11-03

**Authors:** Sithu Bala, Spencer Ellis

**Affiliations:** East and North Hertfordshire Teaching NHS Trust, Stevenage, United Kingdom; East and North Hertfordshire Teaching NHS Trust, Stevenage, United Kingdom

## Abstract

**Introduction:**

Anti-phospholipid syndrome (APS) is a systemic autoimmune disease that can result in recurrent arterial, venous or microvascular thrombosis and obstetric manifestations. Posterior reversible encephalopathy syndrome (PRES) is a clinico-radiological syndrome characterised by acute or subacute onset of neurological dysfunction and bilateral subcortical vasogenic oedema resulting from endothelial dysfunction and blood-brain barrier disruption. Hypertension, renal and autoimmune diseases are recognised risk factors. We present a diagnostically challenging case of acute encephalopathy in a patient with historical triple-positive APS where differentiating PRES from cerebral lupus, infection and thrombotic disease proved challenging.

**Case description:**

46-year-old woman with systemic lupus erythematosus, historical triple-positive antiphospholipid syndrome and end stage renal disease secondary to childhood focal segmental glomerulosclerosis on maintenance haemodialysis presented after being found collapsed at home, having missed scheduled haemodialysis. Information regarding her original APS diagnosis was limited because diagnosis and follow-up had occurred across multiple centres. Historical records documented previous pulmonary embolism and a left middle cerebral artery ischaemic stroke within the preceding five years. Despite these thrombotic events, she was not receiving long-term anticoagulation because of recurrent severe epistaxis and patient’s preference.

On admission she was severely hypertensive (peak blood pressure 211/128 mmHg) and cerebrally obtunded requiring intensive care. Neuroimaging with CT brain, CT venography and MRI brain demonstrated no acute infarction, haemorrhage, mass lesion or venous sinus thrombosis. CSF analysis excluded bacterial, viral and fungal infection. Autoimmune encephalitis antibodies were negative and EEG demonstrated non-specific encephalopathy.

Laboratory evaluation showed a CRP peaking at 131 mg/L with only a marginally elevated ESR (25 mm/hr). In conjunction with normal complement levels and negative anti-double-stranded DNA antibodies, these findings made acute neuropsychiatric lupus less likely, while the disproportionate CRP elevation was atypical for an isolated lupus flare.

Anticardiolipin and anti-B2 glycoprotein-1 antibodies remained persistently positive at low titres, supporting the diagnosis of APS. Lupus anticoagulant testing was uninterpretable because therapeutic heparin had already been commenced. Catastrophic APS was considered unlikely in the absence of multi-organ thrombosis.

Following exclusion of alternative diagnoses, the presentation was considered most consistent with hypertensive encephalopathy/ posterior reversible encephalopathy syndrome (PRES) in the context of severe hypertension, end-stage renal disease and underlying APS. Neurological function improved with blood pressure control, haemodialysis and supportive care. Therapeutic anticoagulation was maintained throughout admission. Given concerns regarding adherence and the patient’s refusal of warfarin, she was discharged on renal-adjusted apixaban following haematology recommendation.

**Discussion:**

This case demonstrates the diagnostic complexity of acute encephalopathy in a patient with historical triple-positive APS, severe hypertension, and dialysis-dependent renal failure. The principal challenge was distinguishing between recurrent thrombotic APS, neuropsychiatric lupus, central nervous system infection, or PRES.

APS was an important diagnostic consideration because the patient was not receiving anticoagulation despite a history of major arterial thrombotic events and historical triple-positive antiphospholipid antibody status. According to revised Sapporo and 2023 ACR/ EULAR APS classification criteria, previous confirmed thrombosis combined with persistent antiphospholipid antibodies fulfil classification requirements for definite APS. Consequently, catastrophic APS and recurrent cerebrovascular thrombosis require urgent exclusion. Normal complement levels, negative anti-ds DNA antibodies, absence of features of active lupus and unremarkable neuroimaging reduced likelihood of cerebral lupus. Extensive microbiological investigations excluded infectious meningoencephalitis.

The diagnosis of PRES was supported by the presence of severe uncontrolled hypertension, missed haemodialysis, uraemia, acute encephalopathy, and subsequent neurological recovery following blood pressure optimisation and renal replacement therapy. Although the relationship between APS and PRES remains unclear, up to 50% of patients with PRES have underlying autoimmune disorders supporting immune-mediated endothelial dysfunction as a potential contributing mechanism.

This case also highlights the challenges of anticoagulation in high-risk APS. Current international guidelines recommend vitamin K antagonist therapy for thrombotic APS, particularly in patients with arterial thrombosis or historical triple-positive antibodies while evidence supporting direct oral anticoagulants (DOACs) remains limited and is not considered a guideline-preferred alternative for high-risk APS.

Anticoagulant selection must also account for patient preferences and treatment adherence. Previous bleeding complications, refusal of warfarin, and concerns regarding long-term compliance precluded guideline-recommended therapy. Following multidisciplinary discussion, renal-adjusted apixaban was selected as an individualised compromise, reflecting clinical judgement that some anticoagulation was preferable to no secondary thromboprophylaxis in a patient with substantial lifetime thrombotic burden.

**Key learning points:**

1. Acute encephalopathy in APS has broad differential diagnosis including PRES, cerebral lupus, CNS infection, uraemic encephalopathy and recurrent thrombotic cerebrovascular disease.

2. PRES should be considered in APS patients presenting with severe hypertension, renal dysfunction, possibly through mechanisms of endothelial dysfunction and microvascular injury dependent of overt thrombosis.

3. Distinguishing PRES from cerebral lupus can be challenging. Clinical context, lupus serology, neuroimaging and multidisciplinary assessment are essential for diagnosis.

4. Severe hypertension and missed haemodialysis were likely important contributors to PRES development in this patient.

5. Multidisciplinary collaboration between specialities was critical in reaching the diagnosis and guiding management.

6. Current international guidance from the European Alliance of Associations for Rheumatology (EULAR), European Society of Cardiology (ESC), American Society of Haematology (ASH), British Society for Haematology (BSH) and International Society on Thrombosis and Haemostasis (ISTH) recommend vitamin K antagonist therapy in patients with thrombotic APS, particularly those with arterial thrombosis or historical triple-positive antiphospholipid antibodies, as direct oral anticoagulants (DOACs) have been associated with an increased risk of recurrent arterial thrombosis in this population.

7. When guideline-recommended anticoagulation is not feasible, anticoagulation selection should be individualised through multidisciplinary shared decision-making, balancing thrombotic risk, bleeding history, treatment adherence and patient preference.

